# Mr. Wilkinson, on Mr. Cuming's Reply to Dr. Scott

**Published:** 1805-02-01

**Authors:** G. Wilkinson

**Affiliations:** Sunderland


### *To the Editors of the Medical and Physical Journal.*

Gentlemen,

THE ardent zeal, and unceasing industry, evinced by Mr. Cuming in pointing out such modes of treatment as he has found successful in practice, is certainly commendable. But the confident, positive, and assuming manner in which he enforces them, together with his impatience of contradiction, and want of temper to those who have ventured to differ in opinion with what he deems his *unerring observation and experience*,* must, whatever may be their importance, certainly render them less palatable than if they were offered in milder terms.

These reflections, which occurred to me on the perusal of his irritable reply † to Dr. Scott's fair, and I think, not uninteresting remarks ‡ on his paper relative to the cure of intermitting fevers, by the use of white vitriol, § were not a little strengthened by the disrespectful manner which in his paper on leprosy, in No. 69, he handles the flour bag || of the late Dr. Cullen.

I shall not enter into a discussion of this case, which he after having, as he says, cured as a general affection, considered it as local, from its continuing in the form of fissures, &c. in the palms of his hands, and which, he says, gave

---

* These are Mr. C's own words, Med. and Phys. Journal. No. 61.

† Idem. ‡ No. 60, p. 129. § No. 58, p. 496.

|| So called by Mr. Cuming, No. 69.

way to the application of a blister. No, for although this treatment, which proved efficacious in this and another instance, beyond all doubt, is as ingenious as it is unusual; yet the propensity or itch of snarling at our old masters, (for it appears that Mr. C. from his own confession, was a disciple of this great man) has become so fashionable, that I cannot help exclaiming, not in the words of Virgil, (which he quotes while castigating Dr. Scott) but in the language of our elegant poet, Alexander Pope, who says,

> "We call our fathers fools, so wise we grow;
> "No doubt, our wiser sons will call us so."

Was it not enough for Mr. Cuming, if he *really* intended nothing further than recommending in a case of erysipelas, which he has related, not a new, but perhaps a much older, and *very common* remedy, viz. Goulard's *aq. veg. min.* which he used *cold* in preference to the flour bag, which last probably he had never tried, without *barking* at the *dead old lion*, and from whose *prey** himself had been nurtured when a young *helpless cub*.†

I advance not these remarks from ill temper, enmity, or disrespect to Mr. C. nor do I think them applicable to him alone, but to every, any, or all those who so uncandidly, or at least unadvisedly, and often unnecessarily, insult our truly respectable medical ancestors; and for what? Why, for certain *minor* errors, not worth naming, or what would have escaped notice in less celebrated characters.

To point out, with becoming respect and liberality, the imperfections, mistakes, or errors of our ancestors, or cotemporaries, is just, fair, and admissible; the former by these means are not vilified, but their faults corrected; and the latter have it in their power either to defend what they may judge right, or to recant what is made appear erroneous.

If I have noticed this error, or rather mark of disrespect of Mr. C. to the memory of his late admirable preceptor, Dr. Cullen, and which might have been avoided by not placing it to his account *particularly*, it is because the flour bag, *as he calls it*, does not appear to be the legitimate offspring of the Doctor, but is only named by him

---

* Alluding to the Doctor's lectures or instructions, which Mr. C. is supposed to have attended.

† That is, a mere suckling in medical science, which most young men are prior to commencing their regular studies.

him casually among various other applications used in erysipelas.*

This propensity of undervaluing for trifling errors the reputation of truly great men, is no new thing; it is a failing attached to many, but more especially those who it frequently happens, are inferior in abilities to the persons they are depreciating.

The most remarkable instance of this sort, is that of the celebrated Dr. Mead, a gentleman more conspicuous for the splendour of his character, and the great wealth he acquired in his profession, than perhaps for any remarkable discoveries made (that I am able to ascertain) in the science of medicine.†

He it was, who so unadvisedly attempted to correct the errors of the great and immortal Sydenham; but his misguided reflections were no less ably repelled by Dr. Thomas Dixon.‡

I would not have it considered that I entertain a *superstitious* veneration for our great medical ancestors, but I would cherish a due *respect* for their intrinsic merits.

This disposition has already been evinced by me, in repelling certain disrespectful insinuations aimed lately by a gentleman (with whom I differed in opinion) at the professional character of the late ingenious and benevolent Dr. Fothergill; § and I am equally disposed, on principles of justice and liberality, to offer my reflections on what seems to be Mr. Cuming's disrespectful, and, I fear, intemperate reply to Dr. Scott, more particularly as the Doctor, from the ungentleman-like manner in which he appears to have been treated, rather than refuted, has forborn to answer.

From a simple statement of facts it appears, that Mr. Cuming || having, I think, very satisfactorily and clearly pointed out the frequent failure of what he terms "this hitherto considered *specific*," the cinchona, in the cure of agues, says, "It must be no small satisfaction (for the practitioner) to know that the materia medica contains a remedy

---

* The application that seems most safe, and which is now most commonly employed, is that of a dry mealy powder frequently sprinkled upon the inflamed parts. Vide First Lines, vol. 2, Art. DCCXI.

† Except that of pressure on the abdomen during the evacuation of the water on tapping for the dropsy, which must be allowed of no small importance. Vide Mead's Works on Dropsy.

‡ Med. Obs. and Inquir. vol. iv.

§ Med. and Phys. Journal, vol. vii.

|| Idem, No. 58, vol. x.

remedy *equally infallible,* and more entitled to the epithet *specific;*" and remarks that, "in ships of war I have often given it under *every type* of the disease, and *never* knew it fail." His method of treatment is, "After the primæ viæ have been thoroughly cleansed by means of an antimonial emetic, I begin by giving the patient two or three grains in the day, during the intervals of the fit in this formula. ℞. *Pulv. zinci vitriol. gr. ij. vel. iij. cons. rosæ q. s. ft. bol. No. iij. capt. æger, j. 2da. vel 3tia. quaq. hora, ut opus fuerit, et augeatur dosis, gr. j. in dies.*" He farther says, "I have never had occasion to give more than five grains in the day." These boluses he *always* orders to be washed down with half a tea cupful of any proper diluent.

From these conclusions advanced by Mr. C. it does not appear that Dr. S. was far wrong in supposing he considered his *zinc vitriol* as a *real specific,* which he has since avowed in his reply to the Doctor, published in No. 61 of the Med. and Phys. Journal; in which are remarks on the same subject by Dr. Tice, who says, "The white vitriol I have employed in a number of instances for the cure of agues with very happy success; but I must confess, from an extensive practice and frequent observation, that it does not possess *any specific quality,* and that not unfrequently it has failed as a remedy, especially in those cases where the constitution had suffered from repeated attacks of the fever." He also, "from repeated trials, prefers the sulphat of copper or blue vitriol, to either the solution of arsenic, or the white vitriol;" and that "the effects of the latter are neither so certain, nor are they sufficiently lasting in inveterate cases, as those derived from the blue vitriol, &c."

He farther says, that, "the word *specific sounds empirical,* and is too generally applied; yet, as the cinchona is so powerful a remedy for intermittent fever, and mercury for venereal infections, some indulgence must be allowed to those who term *them specifics.*" Hence it is obvious, that Dr. Scott's remarks, which appear to be general, are considered by Mr. Cuming as applied to him singly. The former says, "The word *specific* I very much dislike; to *me* the sound is *empirical;*" and the latter replies, "and I can assure him, to *me* his observation appears extremely harsh, and unbecoming;" and so firmly does he appear to be impressed with this, (I charitably hope) unintentional affront, that he is at no small pains to insinuate that the Doctor has equally implicated the *Editors* with himself; and and does not scruple professing "to exculpate both *them*, and *himself*, from an imputation which is as gross as it is indecent." He however assures the Doctor, that "the remarks made in his paper, of No. 58, are the result of *unerring observation and experience*."

In his trifling animadversions on the term *pretty well*, used by Dr. S. he has not only misrepresented his meaning, by supposing it (the white vitriol) cured, when other remedies failed; but says, "he believes the cuprum ammon. has been expunged from *some* pharmacopœias, being considered by men eminent in their profession, as a remedy fraught with the greatest danger, and that he *cannot* find it in the Edinburgh Dispensatory, a book which he (Dr. S.) has quoted respecting the white vitriol. I would ask him, what these *some* pharmacopœias are, in which it was, (except the Edinburgh) from which it has been expunged? and who these eminent men were, who considered it as a remedy fraught with the greatest danger? And how it comes to pass that he *cannot* find it *(cuprum ammon.)* in the Edinburgh Dispensatory, although he takes great care to find *his* remedy the *zinc vitriol*. I will not say, as he insinuates to Dr. S. that *his* judgment may be perverted, or he was guilty of *inattention to the subject*; but I may venture to *think*, Mr. C. looked in there for no other purpose than to view his favourite zinc. vitriol.

I must however remark, that I have carefully examined three editions of this book; one of 1786, another 1791, and the last 1803. In all of them the cuprum ammon. is mentioned; and in the last, as a remedy "in incipient phthisis pulmonalis, intermittent fever, and epilepsy."

Dr. Scott says, "his patients could *never* take the quantity of white vitriol mentioned by Mr. Cuming," and gives a præscription of calx zinci in the quantity of a grain twice a day, but for what purpose does not say; he however adds, that he cannot think it deserves the epithet *specific*, (i. e. the white vitriol, which in this case must be allowed preferable to calx zinci). Before this, he says, he "had a good opinion of it, and thought it answered the intention *pretty well*, but prefers the cuprum ammon." Yet Mr. C. is not only displeased at this, but that "the compilers of this work, (the Edinburgh Dispensatory) who do not appear to have known that zinc. vitriol. possessed the virtue of curing agues per se," (arrogantly exclaims) "and if there is any merit in promulgating to the world a remedy which *stands high in my opinion*, it cannot be the intention tention of the Doctor to detract from it, by laying a claim for originality; a claim which I never presumed to offer; but let Virgil speak for *me.*"

"Haud equidem tali me dignor honore."

Truly, indeed, these transcendently sublime and beautiful *criticisms* are far above my comprehension.

Of what honour then, is Mr. Cuming worthy? Am I to give *him* a claim to the use of nitre or saltpetre applied to *mortifications,* (No. 62, Med. and Phys. Journal), and blisters to the palms of the hands in *lepra,* or such like cutaneous affections, for *originality?*

The former, dissolved in vinegar, has long been successfully used by farriers in the most desperate bruises, and other accidents, even where sphacelus existed, to cows and horses; and the latter has been applied to tetters, herpes, and ring-worms with no small advantage. Nevertheless, should it hereafter appear that both these easily procurable remedies prove equally beneficial in the hands of others, his claim to *originality* in these will scarcely be disputed. When, how, or by whom, the term *specific* was applied to the cinchona and mercury, is of no material consequence. Agues and the venereal disease having long since baffled the most experienced and intelligent practitioners, these two remedies, from their superior efficacy above all others heretofore used in these and such like diseases, acquired *this term,* which, in process of time, has become so very fashionable, that it is not only applied to almost every advertized or quack medicine, but what seems more reprehensible, is used as a very common epithet, not only in the writings of many professional gentlemen to their favourite remedies, but named in the bills and labels of even regular practitioners.

It is but a name, 'tis true; but as this name, term, or epithet, call it what you will, is equally adopted by regulars, as well as irregulars, there surely can be no harm in *any man* saying it is *empirical;* and this last epithet is opposed by another equally as disagreeable I think in *sound* to others, (who if it happen to be used to them, would perhaps give equal offence) called dogmatical, and for any thing we know, even *this,* had it been applied by the Doctor, or any other person, to Mr. C. or his favourite remedy, would have been equally resented.

Hence it appears, that this mighty fuss and offence given by the Doctor to Mr. C. amounts to this. Mr. C. prefers the white vitriol to the cortex, cuprum, and all other remedies, remedies, and says, it deserves the name of *specific*; and to prove this, declares, "*he never knew it fail*," and appeals to his *unerring observation and experience*. Dr. S. equally as much entitled as Mr. C. to appeal to *his attentive observations* on the effects of *these* remedies, says, he has a good opinion of it, but *thinks* the *cuprum ammon. rather* preferable. Yet, because he dislikes the term *specific*, and says the sound is *empirical*, in which Dr. Tice coincides, only allowing it to the cinchona and mercury; it is much to be regretted, that Mr. C. whom I trust has more from the irritated state of his feelings, than malevolence, been led to resent so harshly, what in Dr. S. appears *mere matter of opinion*.

For this cause, and that of Mr. Cuming's cavalier treatment of the truly great and benevolent Dr. Cullen, have I presumed to offer these remarks. They are given, I trust, fairly and candidly; and although they may prove not altogether pleasing, they are by no means intended to excite his enmity, which should I not dread, would nevertheless be sorry to merit. They may however be not uninteresting to the candid admirers of our illustrious medical ancestors; and when this is considered, Mr. C. must, however reluctantly he may consent, allow me the same privilege of censuring in him, what appears perhaps greater improprieties, than those which he so freely has indulged towards others.

I am, &c.

*Sunderland, Nov.* 29, 1804. G. WILKINSON.

